# Improvement of gain and spatial resolution for impulsive stimulated Brillouin scattering microscopy

**DOI:** 10.1016/j.pacs.2025.100696

**Published:** 2025-02-10

**Authors:** Taoran Le, Jiarui Li, Haoyun Wei, Yan Li

**Affiliations:** State Key Laboratory of Precision Measurement Technology and Instruments, Department of Precision Instrument, Tsinghua University, Beijing 100084, China

**Keywords:** Impulsive stimulated Brillouin scattering, Brillouin microscopy, Brillouin gain

## Abstract

Brillouin microscopy has been widely used in the mechanical imaging of cells and tissues, and the signal-to-noise (SNR) ratio limits the spectral integration time. Impulsive stimulated Brillouin scattering (ISBS) microscopy is a new elastic imaging technique. As a variant of stimulated Brillouin scattering (SBS), ISBS can overcome the weak signal of spontaneous Brillouin scattering. A simple model can estimate SBS gain. However, the theoretical ISBS gain has not been compared with SBS gain. This paper gives the theoretical ISBS gain estimation, and experiments are designed to verify estimation reliability. The heterodyne ISBS gain coefficient can be much higher than SBS gain coefficient. The relationship between ISBS gain coefficient and spatial resolution is then discussed. We anticipate that the ISBS setup optimization can improve spatial resolution and gain, potentially enabling fast and high spatial resolution imaging of biological cells.

## 1 Introduction

In recent years, Brillouin microscopy has been greatly developed as a new technology to study the mechanical properties of tissues without label and contact [1], [2], [3], [4], [5]. Mechanical properties play an important role in tissue and cell levels [6], [7], [8], and traditional mechanical mapping technology such as atomic force microscopy [9] and ultrasound elastography [10] cannot meet the requirements of non-contact and high spatial resolution. Scarcelli et al. used the virtually imaged phased array as the spectrometer of the spontaneous Brillouin spectroscopy [1], which greatly shortened the spectral integration time. Spontaneous Brillouin microscopy (SpBM) can reach the submicron resolution combined with the confocal microscopy technique. Now, SpBM has been widely used in biological science and medical research [11], [12], [13], [14], [15], [16], [17], [18], [19].

The low efficiency of spontaneous scattering process, due to low number of thermal phonons, limits the measurement SNR and acquisition rate in SpBM. Ballmann et al. developed stimulated Brillouin microscopy (SBM) [3], which uses two slightly detuned continuous-wave (CW) lasers for phonon excitation. Stimulated phonons can lead to a higher SNR. The integration time of SBM is about 20 ms when the total power is roughly 250 mW [5], [20]. However, if the total power is limited (e.g., below 200 mW), SBM’s SNR will be lower than SpBM’s [21]. The continuous pump and probe can be modulated into pulse mode, and the total average power can be reduced while keeping the SNR constant [21], [22].

Impulsive stimulated Brillouin scattering, a variant of stimulated Brillouin scattering, was originally used to study the ultrafast dynamics of matter [23]. In 2017, Ballmann et al. applied it to Brillouin microscopy and developed impulsive stimulated Brillouin microscopy (ISBM) [4]. The ultrasound generation mechanism in ISBM differs from conventional ultrasound techniques, encompassing two physical origins: (1) photoacoustic effects resulting from light absorption-induced thermal expansion, and (2) electrostrictive forces induced by spatial variations in electric field distribution. The ISBM excites a coherent acoustic field in the sample with two coherent picosecond pulses beams. The acoustic field, as Bragg diffraction grating, modulates the probe beam. ISBM can be divided into homodyne [24], [25], [26] and heterodyne [27], [28], [29], [30]. Homodyne ISBM directly detects the diffracted probe beam while heterodyne ISBM detects the beat between the diffracted probe beam and the reference beam. Homodyne and heterodyne ISBM can significantly shorten the measurement time, and the integration time of a single spectrum can reach sub-milliseconds [25], [30]. How to optimize ISBM parameters for the biological applications remains a fundamental technical question. For example, limited by the optical setup, ISBM has not yet reached the submicron spatial resolution. The mainstream ISBM optical setup consists of grating and 4f system rather than objective lens focusing the beam, and the probe beam size of ISBMs reported so far is larger than 10μm [24], [25], [26], [27], [28], [29], [30].

Experimentally, optimizing pump and probe parameters can improve the acquisition rate and spectral resolution [26], [30], [31], which can be explained by ISBM model. The theoretical model of ISBS was developed in the early ISBS studies [23], [32], [33], [34]. The analytical solutions of acoustic field and Bragg grating diffraction efficiency are given in the model. However, the estimated diffraction efficiency has not been calculated and compared with experimental values.

In this paper, we give a model that describes the relationship between the diffraction efficiency and various parameters. We rewrite the diffraction efficiency as gain and compare the theoretical ISBS gain with SBS gain. The theory shows that ISBS gain is similar to SBS gain, and ISBS gain coefficient can be much higher than SBS gain coefficient. We put forward a model which is suitable for grating-4f heterodyne ISBM and design experiments to verify the accuracy of the gain estimation and find that the gain coefficient is inversely proportional to the magnification of the 4f system.

Finally, we discuss the relationship between spatial resolution and gain coefficient based on the above theory and experiment. For ISBM, the shorter the acoustic wavelength (which allows a higher spatial resolution), the higher the gain coefficient. We built a system with probe beam size of 7.6 μm and demonstrated its ability to image water and Polydimethylsiloxane (PDMS) at low power. We discuss further optimization to ISBM, which allows for sub-micron spatial resolution and stronger signal strength.

## 2 Principle and theory

ISBS consists of two physical processes [24]: (1) the coherent acoustic field is generated by pump pulses excitation; (2) the interaction between the probe beam and acoustic field generates the diffracted probe beam. In this section, the analytical solutions describing the elastic strain and diffraction efficiency under ideal conditions are given and the physical quantities in the expressions are explained.

**Fig. 1:**
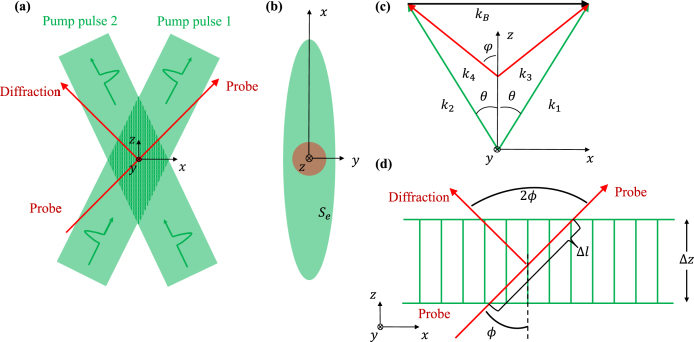
The green beams represent the pump beams and the red beams represent the probe beams. (a) Two coherent pump pulses modulate the sample through electrostriction and photothermal effects in the interference region, and the probe beam is diffracted when it passes through the region. (b) The focal plane of the pump and probe beams in the xy-plane. (c) The coordinate system and pump, probe beams vectors in ISBS. (d) Bragg diffraction.

### 2.1 Acoustic field generation

The two pump pulses cross at a certain angle, and the overlapping area produces an interference pattern shown in Fig. 1(a). The pump pulses excite elastic strains through photothermal and electrostriction effects. The former comes from the crossed pump pulses interference pattern absorption, rapid radiationless relaxation, and periodic temperature distribution. The latter is a consequence of the direct coupling of the laser pulse field to the material’s acoustic field [35]. To calculate the light-induced change of relative dielectric constant. We use the heat conduction and Navier–Stokes equations [32], [33] to describe the stimulated Brillouin scattering. The equation and the specific solving process can be found in Supplementary materials. The change of relative dielectric constant δɛ is:

(1) $$\delta ɛ\left(r,t\right)=\frac{\gamma_{e}\pi I_{pump}}{ncv\Lambda \rho_{0}S_{e}}L\left(t\right)\cos\left(k_{B}\cdot r\right)={ɛ}_{1}\left(t\right)\cos\left(k_{B}\cdot r\right)\text{.}$$

The result is consistent with Ref. [35], and the relative dielectric constant is periodically modulated in a certain region. ${ɛ}_{1}$ is the modulation amplitude. $\gamma_{e}$ is the electrostriction constant expressing the coupling magnitude between light and elastic strain through the electrostriction effect. n is the refractive index and c is the light speed in vacuum. v is the low-frequency limit of the speed of sound. $I_{pump}$ is the two pump pulses energy sum. $k_{B}$ is an acoustic wave vector as shown in Fig. 1(c). $k_{B}=\left|k_{B}\right|$ is the wave number. r is the coordinate vector of the sample coordinate system. $\Lambda =2\pi /k_{B}$ is the phonon wavelength. $\rho_{0}$ is the average density. $S_{e}$ is the pump beam area on the sample. L(t) is a response function and is expressed as [35]:

(2) $$L\left(t\right)=-\gamma_{a}\left[\exp\left(-\Gamma_{R}t\right)-\exp\left(-\Gamma_{b}t\right)\cos\left(\omega_{b}t\right)\right]+\gamma_{e}\exp\left(-\Gamma_{b}t\right)\sin\left(\omega_{b}t\right)\text{.}$$

$\gamma_{a}$ expresses the contribution of the photothermal effect to the dielectric change and is expressed as: $\gamma_{a}=2nc\alpha v^{2}\beta /\left(C_{p}\omega_{b}\right)$. $\Gamma_{b}=\eta k_{B}^{2}/\left(2\rho_{0}\right)$ is the damping constant of the phonon. $\omega_{b}={\left(k_{B}^{2}v^{2}-\Gamma_{b}^{2}\right)}^{1/2}$ is the angular frequency. $\Gamma_{R}=\lambda k_{B}^{2}/\left(\rho_{0}C_{v}\right)$ is the temperature relaxation rate. Usually, $\Gamma_{b}\gg \Gamma_{R}$, which indicates that the spatially periodic temperature distribution, i.e., thermal grating, remains after the propagation of the phonon and then becomes obscure by the heat diffusion. α, β, η, and λ are the absorption coefficient of the light by the material, thermal expansion coefficient, viscosity, and heat conductivity, respectively. $C_{p}$ and $C_{v}$ are the specific heat at constant pressure and constant volume.

The first and second terms of Eq. (2) correspond to the photothermal and electrostriction contributions. The photothermal term is characterized by the coexistence of the relaxational term and the damped harmonic oscillation. For room temperature water, β = 0.00021/K, λ = 0.6 W/(m K), v = 1500 m/s, $C_{p}$ = 4180 J/(kg K), $\gamma_{e}$ = 0.82. For visible pump laser, $\alpha <10^{-2}$/cm, it can be calculated that $\gamma_{a}\ll \gamma_{e}$. The electrostriction effect is dominant in this case. If pump laser is near-infrared, the photothermal effect may not be ignored. Ignoring the heat accumulation requires $\Gamma_{R}$ to be much smaller than the repetition frequency of the pump pulse. In the reported experiments [25], [26], when the photothermal effect cannot be ignored, a high repetition rate may result in heat accumulation affecting the relative dielectric constant. The heat accumulation effect is not considered in this model.

### 2.2 Bragg diffraction

Suppose the probe laser is a CW laser. The volume of the acoustic field stimulated by the pump pulses is much larger than the detected volume by the probe beam and the probe beam size is larger than the phonon wavelength. So the probe beam undergoes a Bragg diffraction in the acoustic field. The diffraction efficiency is given by [34]:

(3) $$\eta_{B}\left(t\right)=\sin{\left[\frac{\pi \Delta z{ɛ}_{1}\left(t\right)}{2n\lambda_{probe}\cos\phi }\right]}^{2}\approx {\left[\frac{\pi \Delta l{ɛ}_{1}\left(t\right)}{2n\lambda_{probe}}\right]}^{2}\text{.}$$

Δz is the interaction length of the probe beams in the z direction. As shown in Fig. 1(d), the effective interaction length Δl is expressed as: Δl=Δz/cos(ϕ). $\lambda_{probe}$ is the wavelength of the probe laser. ϕ is the incidence angle of probe beam.

For SBS, the Brillouin gain for the probe beam is given by [21]:

(4) $$G_{B}\left(\omega_{b}\right)=g_{0}\frac{P_{pump}\Delta L}{S_{p}}\text{,}$$

(5) $$g_{0}=\frac{4\pi^{2}\gamma_{e}^{2}}{n\lambda_{probe}^{2}\rho_{0}vc\Gamma_{B}}\text{.}$$

$P_{pump}$ is the total pump power. $S_{p}$ is the pump beam area and ΔL is the effective acousto-optic interaction length. $g_{0}$ is the Brillouin gain factor. $g_{0}\Delta L/S_{p}$ is the Brillouin gain coefficient. $\Gamma_{B}$ is the damping constant of backscattered phonons. For water ($\Gamma_{B}/2\pi \approx$ 350 MHz), we get $g_{0}=3.5\times 10^{-5}\mu$m/W at $\lambda_{probe}$ = 780 nm [21]. Introducing Eqs. (2), (5) into Eq. (3), we get:

(6) $$\eta_{B}\left(t\right)={\left[\frac{\pi^{2}\gamma_{e}^{2}P_{pump}\Delta l}{2n^{2}\lambda_{probe}\Lambda \rho_{0}vcFS_{e}}L\left(t\right)\right]}^{2}={\left[g_{0}\cdot \frac{\lambda_{probe}\Gamma_{B}}{8n\Lambda F}\cdot \frac{P_{pump}\Delta l}{S_{e}}\exp\left(-\Gamma_{B}t\right)\sin\left(\omega_{b}t\right)\right]}^{2}\text{.}$$

$I_{pump}=P_{pump}/F$. F is the pump pulse repetition rate. By comparing Eq. (6) with Eq. (4), we can find that both expressions include Brillouin gain factor $g_{0}$, pump power, acousto-optic interaction length, and pump beam area. So we call $\sqrt{\eta_{B}\left(t\right)}$ as ISBS gain.

Eq. (6) has an additional term $\lambda_{probe}\Gamma_{B}/\left(8n\Lambda F\right)$. When Λ is 4.13μm and F is 10 kHz, the value is about 3900, much larger than 1. The shorter the acoustic wavelength, the higher the diffraction efficiency. The electrostrictive force per unit volume is related to the spatial frequency of relative dielectric constant and is inversely proportional to the phonon wavelength. Intuitively, the higher the spatial frequency of the relative dielectric constant (or refractive index), the greater the electrostrictive force per unit volume. That is why $\lambda_{probe}$ and phonon wavelength Λ appear in the numerator and denominator, respectively. In SBS, the phonon wavelength is $\lambda_{probe}/2n$.

$F/\Gamma_{B}$ is the phonon duty cycle (the ratio of the excited phonon’s duration time to the pulse interval time). $1/\Gamma_{B}$ can estimate the lifetime of excited phonons under a single pulse, and 1/F is the pump pulse interval time. In the case of constant average power, the lower the duty cycle, the stronger the signal. In pulsed SBM, if pumps are modulated as pulses (assuming that the probes in ISBM and pulsed SBM are still continuous waves). The signal is inversely proportional to the duty cycle. Recently, the duty cycle of pulsed SBS can be as low as 0.05 [21], [22]. The duty cycle of ISBS can be less than 10^−5^ (repetition frequency F = 10 kHz).

Generally, the optical focal volume of pump beam is larger than that of probe beam, which makes $\Delta l/S_{e}$ smaller than $\Delta L/S_{p}$. If pump beam and probe beam have close size, $\Delta l/S_{e}$ and $\Delta L/S_{p}$ are of the same order of magnitude. For heterodyne ISBM, the signal strength is proportional to the square root of $\eta_{B}\left(t\right)$.

### 2.3 Signal-to-noise ratio

We use the same method as [21] to analyze the SNR of homodyne ISBM and heterodyne ISBM. It is assumed that shot noise is dominant and time resolution of the detect system is sufficient to distinguish the relaxation oscillation.

For homodyne ISBM, the SNR is:

(7) $$SNR_{1}\left(t\right)=\frac{{\left[\int_{0}^{\Delta t}\eta P_{probe}dt\right]}^{2}}{\int_{0}^{\Delta t}\eta P_{probe}dt}=\left[g_{0}\cdot \frac{\lambda_{probe}\Gamma_{B}}{8n\Lambda F}\cdot \frac{P_{pupmp}\Delta l}{S_{e}}\right){\left(\times \;\exp\left(-\Gamma_{B}t\right)\sin\left(\omega_{b}t\right)\right]}^{2}P_{probe}\Delta t\text{.}$$

$P_{probe}$ is the probe power and $\Delta_{t}$ is the time resolution or the integration time, $\Delta t\ll 1/\omega_{b}$. For heterodyne ISBM, the interference signal S can be written as [29]:

(8) $$S\left(t\right)=2\sqrt{\eta_{B}\left(t\right)}P_{probe}\text{.}$$

For heterodyne ISBM, the SNR is:

(9) $$SNR_{2}\left(t\right)=\frac{{\left[\int_{0}^{\Delta t}2\sqrt{\eta }\left(t\right)P_{probe}dt\right]}^{2}}{\int_{0}^{\Delta t}P_{probe}dt}=4\left[g_{0}\cdot \frac{\lambda_{probe}\Gamma_{B}}{8n\Lambda F}\cdot \frac{P_{pupmp}\Delta l}{S_{e}}\right){\left(\times \;\exp\left(-\Gamma_{B}t\right)\sin\left(\omega_{b}t\right)\right]}^{2}P_{probe}\Delta t\text{.}$$

ISBM detects the relaxation oscillation in the time domain, while SBM scans the pump and probe frequency difference in the frequency domain. The SNR of SBS is

(10) $$SNR_{3}\left(\omega_{b}\right)=\frac{{\left(\int_{0}^{\Delta t^{\prime }}GP_{probe}dt\right)}^{2}}{\int_{0}^{\Delta t^{\prime }}P_{probe}dt}={\left[g_{0}\cdot \frac{P_{pupmp}\Delta L}{S_{p}}\right]}^{2}P_{probe}\Delta t^{\prime }\text{.}$$

$\Delta t^{\prime }$ is the single point integration time in SBS. Assume $\Delta t=\Delta t^{\prime }$. If the pump beam size and acousto-optic interaction length of ISBS and SBS are the same, the peak SNR of ISBS is much higher than that of SBS.

If the ISBM time resolution is insufficient to distinguish the relaxation oscillation, the SNR will decrease. In the worst case where the entire relaxation oscillation is captured within the integration time, the signal will be proportional to the total energy of the relaxation oscillation.

### 2.4 Spatial resolution

In SBM, the pump and probe beams focus on the sample through the objective lens and the focal size approaches the diffraction limit. $S_{p}$ and ΔL in Eq. (4) can be expressed by numerical aperture NA of the objective lens and optical wavelength [21]. For an ideal Gaussian light, $\Delta L/S_{p}$ is about $2n/\lambda_{probe}$. In mainstream ISBM, the pump and probe beams focus on the sample through the grating-4f system, so $S_{e}$ and Δl cannot be expressed by NA. It is necessary to establish the relationship between the grating-4f system and the focal size. The following takes heterodyne ISBM as an example.

**Fig. 2:**
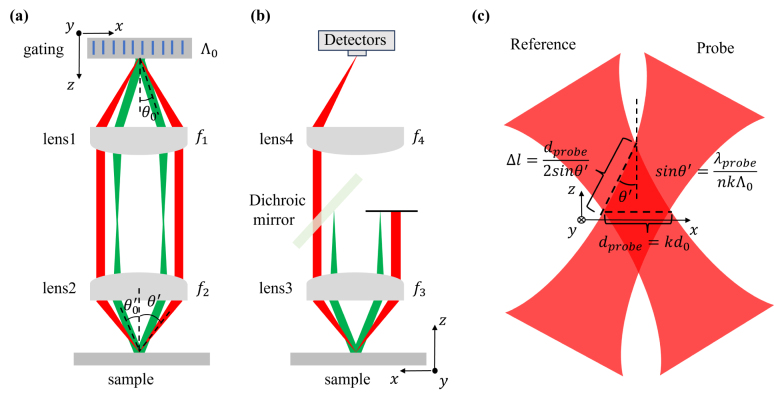
The green beam represents the pump beam and the red beam represents the probe beam. (a) grating-4f system in ISBM. (b) a second 4f system collects diffracted probe beam and reference beam passing through the sample. (c) probe beam and reference beam overlap at the detection volume.

Fig. 2(a) shows the grating-4f system, the grating period is $\Lambda_{0}$, and the focal lengths of the two lenses are $f_{1}$ and $f_{2}$. The diffraction angle of the pump beam and the angle of the pump beam incident on the sample are given by:

(11) $$\sin\theta_{0}=\frac{\lambda_{pump}}{\Lambda_{0}}\text{,}$$

(12) $$\sin{\theta_{0}}^{\prime }=\frac{f_{1}}{f_{2}}\sin\theta_{0}=\frac{\sin\theta_{0}}{k}\text{.}$$

$k=f_{2}/f_{1}$ is the 4f magnification. Acoustic wave number $k_{B}$ and wavelength Λ are:

(13) $$k_{B}=2k_{1}\sin{\theta_{0}}^{\prime }=\frac{f_{1}}{f_{2}}\frac{4\pi }{\Lambda_{0}}\text{,}$$

(14) $$\Lambda =\frac{2\pi }{k_{B}}=\frac{k}{2}\Lambda_{0}\text{.}$$

The 4f system images the spot on the grating onto the focal plane with a k magnification. As shown in Fig. 2(a), the incident beams of the grating-4f system are a y-direction collimated pump beam and a focused probe beam. The transmitted beams of the grating-4f system are two y-direction collimated pump beams and two focused probe beams. Focal plane is in the sample. The incident pump beam is set up to produce extended interference fringes on the focal plane, which help accurately calibrate sample viscosity and improve spectral resolution [31]. The focused probe beams produce a small probe volume. The sizes do not approach the sub-cellular resolution, regardless of pump or probe beams. The relationship between the pump beam area $S_{e}$ on the focal plane and the pump beam area $S_{0}$ on the grating is as follows:

(15) $$S_{e}=k^{2}S_{0}\text{.}$$

The relationship between the probe beam size $d_{probe}$ on the focal plane and the probe beam area $d_{0}$ on the grating is as follows:

(16) $$d_{probe}=kd_{0}\text{.}$$

$S_{e}$ and $d_{probe}$ can be calculated by a camera, and $S_{0}$ and $d_{0}$ are determined by the setup before the grating-4f. In heterodyne ISBS, diffracted probe beam overlapping with the reference beam interferes at the detector to produce the beat signal, while diffracted probe beam at other locations does not.

This can be illustrated in Fig. 2(b). Passing the sample, pump beams, probe beam, reference beam and diffracted probe beam enter a second 4f system, where pump beam and probe beam in one way are blocked, and pump beam in the other way is blocked by a dichroic mirror. The remaining reference beam and diffracted probe beam are focused on the detector, and the interference signal is generated by the overlapping part at the focal point in the sample. Fig. 2(c) shows the overlap of the probe and reference beam at the focal point, and the overlapping volume is the detection volume. According to the geometry in Fig. 2(c), overlapping length Δl is:

(17) $$\Delta l=\frac{d_{probe}}{2\sin\theta^{\prime }}\text{.}$$

$sin\theta^{\prime }=\lambda_{probe}/nk\Lambda_{0}$, Eq. (17) becomes:

(18) $$\Delta l=\frac{nk^{2}d_{0}\Lambda_{0}}{2\lambda_{probe}}\text{.}$$

Δl and $S_{e}$ are proportional to $k^{2}$, so $\Delta l/S_{e}$ is a constant. Based on the transverse size of the probe spot, the corresponding Rayleigh length of probe beam can be obtained as follows:

(19) $$z_{R}=\frac{nk^{2}\pi {d_{0}}^{2}}{4\lambda_{probe}}\text{.}$$

An effective diffraction of probe beam through the grating requires $d_{0}$ greater than $\Lambda_{0}$, so $z_{R}$ is greater than Δl. Therefore, on the Δl dimension, probe beam can be regarded as collimated beam, and Eq. (3) is still applicable. Probe beam undergoes Bragg diffraction. After leaving the focal region, the diffracted beams diverge, just as the undiffracted beam, because the diffracted beams have the same diameter as the undiffracted beam and differ from the incident beam only in amplitude and propagation direction.

Introducing Eqs. (15), (18), the Eq. (8) becomes:

(20) $$S\left(t\right)=g_{0}\cdot \frac{\Gamma_{B}}{4F}\cdot \frac{P_{pupmp}d_{0}}{kS_{0}}\cdot \exp\left(-\Gamma_{B}t\right)\sin\left(\omega_{b}t\right)\cdot P_{probe}=CP_{pupmp}P_{probe}\exp\left(-\Gamma_{B}t\right)\sin\left(\omega_{b}t\right)$$

$C=g_{0}\Gamma_{B}d_{0}/\left(4kFS_{0}\right)$ is the heterodyne ISBM gain coefficient. In SBM, the gain coefficient is approximately $1.0\times 10^{-4}$ /W for water [21].

The gain of heterodyne grating-4f ISBS links the heterodyne interference intensity with the sample parameters and the optical parameters. The gain can be improved by a smaller k which also improves spatial resolution. This result is as expected because the phonon wavelength is proportional to k and $\Delta l/S_{e}$ is independent of k. Limited by lens focal length and size, k cannot be infinitely small.

## 3 Methods

### 3.1 Experimental setup

**Fig. 3:**
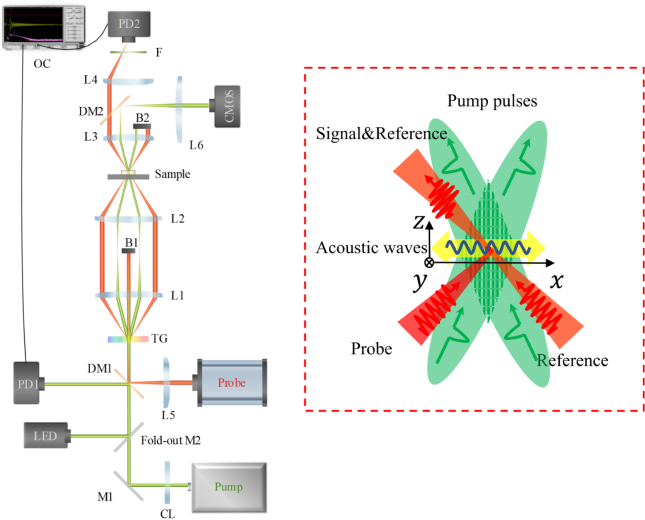
Setup of ISBS microscope. Pump: Pump laser. Probe: Probe laser. DM1,DM2: dichroic mirror. M1,M2: mirror. TG: transmission grating. L1, L2, L3 and L4: achromatic lenses. CL: cylindrical lens. L5,L6: spherical lenses. B1,B2: block. F: 780 nm bandpass filters. PD1,PD2: photodetector. OC: oscilloscope. LED: green LED. CCD: CCD camera. The 4f system is vertical in space and the sample is placed horizontally on a displacement platform. Inset describes the excitation and detection of acoustic waves.

The setup and principle of the heterodyne ISBS microscope are shown in Fig. 3. We choose a 532 nm ultra-fast laser (10ps, Huaray Laser, PINE-532-15) as the pump laser, and a 780 nm continuous laser (Thorlabs, DBR780PN) with a tapered amplifier as the probe laser. In the experiment, the pump repetition rate is 10 kHz. Pump beam and probe beam are focused on the grating (HOLO OR, DS-278-Q-Y-A, with a grating period of 20.7 μm). The patterns of the pump and probe beams on the focal plane are shown in the inset of Fig. 3. Two pump beams interfere on the focal plane to form an elliptical-like pattern. The probe spot is located in the center of the interference area with the biggest acoustic modulation. The focal plane of pump beam coincides with the largest plane in the transverse overlap region in the axial direction. Acoustic wavelength Λ matches the interference pattern. The measurement plane coincides with the focal plane of the probe and reference beam. Elastic acoustic waves are excited in the interference area, forming a standing wave field in a uniform medium. The standing wave forms a time-dependent grating. Probe beam interacts with the acoustic wave generating diffracted probe beams. The diffracted beam and the reference beam are in the same direction and spatially coincident. A photodetector PD1 is used to generate a trigger signal. The heterodyne ISBS signal is detected by a photodetector PD2 (New Focus, 1601FS-AC), and then passes through two-stage cascade amplifiers (Mini Circuits, ZFL-1000LN+, 1 GHz). The time-domain signal is acquired and stored by a high-sampling-rate oscilloscope (Agilent Technologies, DSO9254 A). A green LED (daheng optics, GCI-0604) combined with a CMOS camera (Thorlabs, CS126MU) allows for brightfield imaging.

The photodetector and power amplifier determine the conversion factor from optical power to the measured voltage of the photoelectric detection system, which is about 35000 V/W in our setup. The total power of the pump pulses on the sample ranges from 31 mW to 116 mW. The probe laser power is constant, the reference and probe power sum on the sample is 4.7 mW, and the power on the detector is 2 mW. The sample is located on a high-precision scanning stage (Thorlabs, MLS203-1) to scan in the xy-plane. The scanning in the z-direction is achieved by a motorized translation stage (Thorlabs, ZFM2020) to move the grating-4f. The scanning stage and oscilloscope are synchronized and controlled by homebuilt LabVIEW scripts.

To get higher spatial resolution, the second lens $L_{2}$ in the 4f system can be replaced with an objective lens (Olympus, PLN 10X), note that this is only due to the short focal length of the objective lens. We do not fill the aperture of the objective lens with an expanded probe beam.

### 3.2 Data processing

We use the Matrix pencil (MP) method to process data. MP is suitable for processing time-domain relaxation oscillation data [36]. Compared with the Fast Fourier transform (FFT), MP method can reduce spectral leakage and spectral broadening caused by window function [28], [36]. MP has stronger anti-noise ability than FFT.

The relaxation oscillation signal measured can be expressed as:

(21) $$S\left(t\right)=A\exp\left(-\alpha t\right)\sin\left(2\pi f_{0}t\right)\text{.}$$

MP can extract the initial peak amplitude A, frequency $f_{0}$, and attenuation coefficient α of relaxation oscillation. The corresponding spectrum function is

(22) $$\overset{ˆ}{S}\left(f\right)=\frac{A}{{\sqrt{\alpha^{2}+\left[2\pi \left(f-f_{0}\right)\right]}}^{2}}\text{.}$$

The linewidth of the peak in the frequency spectrum is

(23) $$\Delta f=\frac{\sqrt{3}\alpha }{\pi }\text{.}$$

## 4 Results and discussion

### 4.1 Pump and probe beams sizes

**Fig. 4:**
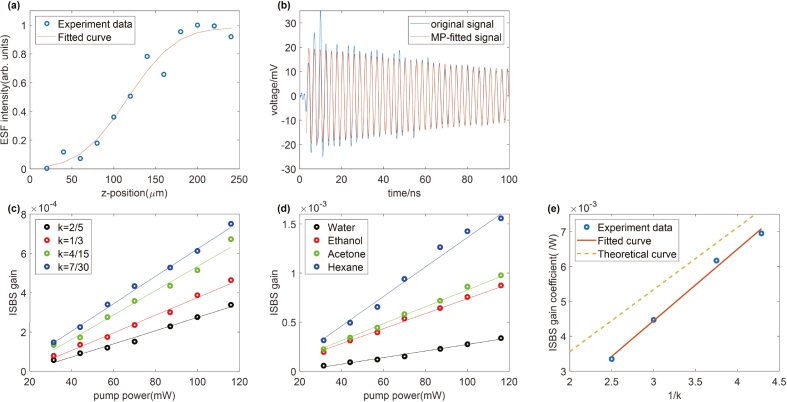
(a) Rescaled ESF measured along the z-axis. (b) Water phonon relaxation oscillation with the pump power of 116 mW. (c) ISBS gain curves at different k. (d) ISBS gain curves for different reagents. (e) The gain coefficients at different k.

To determine the value of $d_{0}/S_{0}$, we measured $d_{probe}$ and $S_{e}$ at k=0.4. The pump beam is focused on the focal plane with an area of 472 μm × 16 μm, which is measured by a beam quality analyzer (Spiricon, BGS-USBSP620). The probe beam size $d_{probe}$ achieves 14 μm. From Eq. (18), (19), overlapping length Δl is about 100 μm and the Rayleigh length $z_{R}$ is about 262 μm for PDMS. To obtain the interaction length, PDMS with smooth surfaces are used as sample and the grating-4f system is scanned along the z-axis, from the air to the PDMS sample. The signal strengths under different z coordinates are recorded(Fig. 4(a)). The edge spread function (ESF) function fits the signal strength curve, giving a z-axis interaction length of about 107 μm. The theoretical overlapping length Δl is close to the experimental interaction length, so the overlapping length is more accurate for estimating the interaction length than the Rayleigh length.

### 4.2 Heterodyne gain measurement

Scanning pump power, a curve of ISBS gain and pump power can be obtained. We use deionized water as the standard substance and the magnification k is 0.4. The signal amplitude is extracted by MP, as shown in Fig. 4(b). In Fig. 4(c), each point represents the heterodyne ISBS gain at corresponding pump power. The slope, representing the heterodyne ISBS gain coefficient C, is 0.0033/W, which is larger than that of SBM. From Eq. (20), the theoretical peak ISBS gain coefficient is 0.0045/W. Experimental and theoretical gain coefficients are of the same order of magnitude.

**Table 1:** Table of ISBM gain coefficients and ratios of experimental gain coefficient to theoretical gain coefficient for different reagents.

Reagent	Experimental gain coefficient (/W)	Theoretical gain coefficient (/W)	Ratio
Water	0.0033	0.0045	0.73
Ethanol	0.0076	0.0103	0.74
Acetone	0.0086	0.0108	0.80
Hexane	0.0152	0.0194	0.78

**Table 2:** Table of ISBM gain coefficients and ratios of experimental gain coefficient to theoretical gain coefficient for different magnifications.

Magnification	Experimental gain coefficient (/W)	Theoretical gain coefficient (/W)	Ratio
2/5	0.0033	0.0045	0.73
1/3	0.0045	0.0053	0.85
4/15	0.0062	0.0067	0.93
7/30	0.0070	0.0076	0.92

To show the universality of Eq. (20), we select other analytical reagent as the samples and obtain the gain coefficients by the same method, as shown in Fig. 4(d) and Table 1. The ratio of the experimental and theoretical gain coefficients ranges from 0.73 to 0.8. The density, speed of sound and elasto-optic coefficient of the reagent can be found in Ref. [29] and handbook [37].

To verify that there is a predictable linear relationship between the gain coefficient and the reciprocal of 4f magnification k, we use three more sets of 4f systems with k of 1/3, 4/15, and 7/30. As shown in Fig. 4(c) and (e), the smaller k, the larger the slope of the curve. The gain coefficient and 1/k are linearly fitted, and the fitted slope is (0.00205+0.00053)/W. The theoretical slope is 0.00178/W, which is within the 1σ range of the fitted slope. Table 2 shows the gain coefficients.

It is seen that Eq. (20) can estimate the magnitude of gain coefficient for different reagents and magnifications. The ratio of experimental value to theoretical value ranges from 0.73 to 0.93. The factors that cause the difference between the experimental and theoretical values may be the optical phase mismatch, the density fluctuation noise, and the potential defects of the theoretical model.

### 4.3 ISBS spatial resolution

The horizontal spatial resolution of the grating-4f system ISBS system is proportional to k, k is the focal length ratio of the two lenses of the 4f system. Reducing k requires $f_{1}$ to be smaller and $f_{2}$ to be larger. There are tradeoffs between the focal length and lens diameter. For example, the focal length of commercial achromatic lenses with a one-inch diameter is usually greater than 30 mm. To obtain a smaller focal length $f_{1}$, the diameter of lens $L_{1}$ should become smaller. The diameter of $L_{1}$ limits the focal length $f_{2}$, because the longer the focal length $f_{2}$, the farther the pump and probe beams are from the center axis of the lens $L_{1}$. Excessive focal length $f_{2}$ makes lens $L_{1}$ unable to receive pump and probe light.

**Fig. 5:**
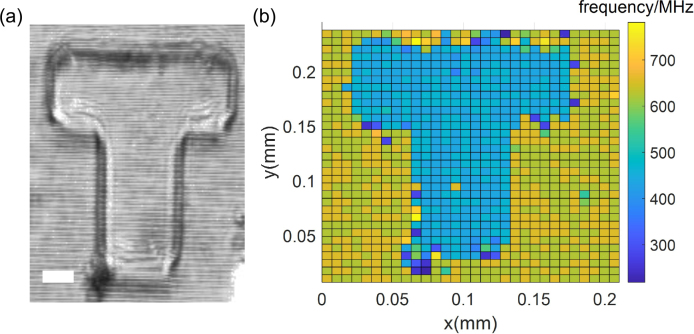
(a) PDMS bright field imaging of T-shaped bulge in the water environment. The scale bar is 25 μm. (b) Brillouin frequency map corresponding to (a).

Here we present a choice of 4f systems, $L_{1}$ is an achromatic double adhesive lens with 80 mm focal length, $L_{2}$ is an Olympus objective with 18 mm front focal length. The objective lens is selected because of its short focal length and can eliminate spherical aberration. In this combination, probe and pump beams are located at the aperture edge of the objective lens. k is 0.225, and the lateral probe beam size is about 7.6 μm.

**Fig. 6:**
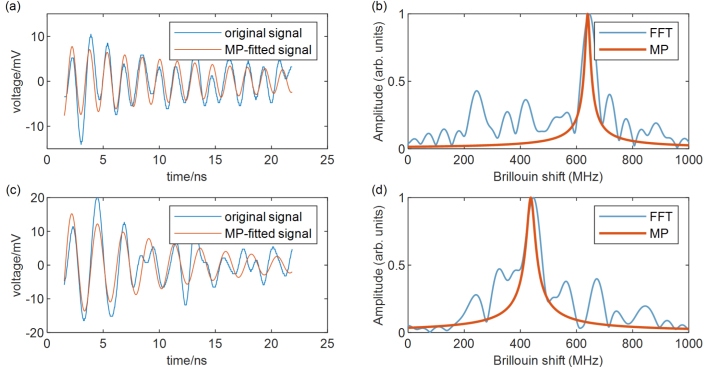
(a) The original signal and MP-fitted signal of water. (b) The FFT and MP of (a). (c) The original signal and MP-fitted signal of PDMS. (b) The FFT and MP of (c).

ISBS imaging is demonstrated with a water-filled PDMS sample (Fig. 5(a)). The PDMS pipe is about 60 μm. The total pump power is 31 mW, and the reference and probe power sum is 4.7 mW, the total power is lower than the reported ISBS microscope [26], [30]. We perform mechanical imaging in the xy-plane with a single pixel integration time of 6.4 ms (64 averaging times). The distribution of Brillouin frequency shift for the sample is mapped in Fig. 5(b) with a total pixel number of 30 × 35 and each pixel size of 7 μm. The mechanical properties of the two materials can be distinguished. On the boundary, because of phase mismatch, information cannot be extracted from some pixels. Fig. 6 (a) and (c) shows the phonon relaxation oscillation of water and PDMS with Brillouin shifts of 640 MHz and 437 MHz respectively. Fig. 6 (b) and (d) are the spectra obtained by FFT and MP. Before processing the data with FFT, we add zeros to the end of the data to smooth out the spectrogram. Compared with FFT, MP can extract spectral information more accurately.

### 4.4 Discussion

The grating-4f system can make the pump and probe satisfy the phase matching in the ISBS process. However, this is the reason for the low optical spatial resolution of ISBS. According to Eq. (6), enhancing the signal requires short acoustic wavelengths and reducing the size difference between the pump and probe beams. The acoustic wavelength is determined by the angle between the two pump beams. In the grating-4f system, the angle cannot reach 180 degrees and the acoustic wavelength cannot get the minimum value. The phonon mean free path limits pump spot size. If the transverse length of the pump spot is shorter than the phonon mean free path, the spectral resolution will decrease. One possible way to improve spatial resolution and gain is to abandon the grating-4f system. Four objective lenses focus four beams on the sample. Two pump beams propagate in opposite directions. The probe and reference beams are incident at angles satisfying Bragg diffraction. The probe’s objective lens determines the spatial resolution. The phonon wavelength excited by the opposite propagating pump is the shortest. The propagation direction of phonons is the same as that of pump beams, so the size of pump spot does not affect the spectral resolution. The size difference between pump and probe beams can be reduced. The advantages of a similar optical setup are discussed in Ref. [26]: high spectral resolution, short phonon mean free path. Both spatial resolution and gain can be improved.

## 5 Conclusion

In this paper, we summarize and reorganize the theoretical model of ISBM. The analytical solution of gain in heterodyne ISBM is given. It can be proved that with the same optical power density and optical volume, ISBM can have higher gain and SNR than SBM. The experimental gain of ISBM coefficient is the same order of magnitude as the theoretical value. We prove that the gain coefficient is inversely proportional to the magnification of the 4f system. A lower magnification can be obtained by replacing a lens in the 4f system with an objective lens. Experiments show that ISBS can distinguish well water from PDMS with low power.

## CRediT authorship contribution statement

**Taoran Le:** Writing – review & editing, Writing – original draft, Validation, Methodology, Formal analysis, Data curation, Conceptualization. **Jiarui Li:** Writing – review & editing, Methodology, Conceptualization. **Haoyun Wei:** Writing – review & editing, Project administration. **Yan Li:** Writing – review & editing, Supervision, Project administration, Funding acquisition.

## Declaration of competing interest

The authors declare that they have no known competing financial interests or personal relationships that could have appeared to influence the work reported in this paper.

## Data Availability

Data will be made available on request.

## Appendix A Supplementary data

MMC S1 Due to electrostriction and photothermal effects, the relative dielectric constant in the sample is modulated.
