# Supplementary material for: Improvement of gain and spatial resolution for impulsive stimulated Brillouin scattering microscopy
Source: Photoacoustics. 2025 Feb 10;42:100696. doi: 10.1016/j.pacs.2025.100696 (PMC11874816; doi:10.1016/j.pacs.2025.100696)
Supplement: MMC S1 — Due to electrostriction and photothermal effects, the relative dielectric constant in the sample is modulated. [file mmc1.docx]

**Supplement: Improvement of gain and spatial resolution for impulsive stimulated Brillouin scattering microscopy**

**Taoran Le,**1 **Jiarui Li,**1 **Haoyun Wei,**1 **and Yan Li**^1^, a)

[1](#_bookmark8)State Key Laboratory of Precision Measurement Technology and Instruments, Department of Precision Instrument, Tsinghua University, Beijing 100084, China

[a)](#_bookmark8)Author to whom correspondence should be addressed: [liyan@mail.tsinghua.edu.cn](mailto:liyan@mail.tsinghua.edu.cn)

**Principle of the impulsive stimulated Brillouin scattering.**

The conservation rules of mass, momentum, and energy generally express the state of the material under the external light field. In liquids, these relations become simplified into the following two equations concerning the changes in density and temperature [1,2]:

$\frac{\partial^{2}\rho}{\partial t^{2}}-\frac{v^{2}}{\gamma}\Delta\rho-\frac{\eta}{\rho_{0}}\Delta\frac{\partial\rho}{\partial t}-\frac{v^{2}\beta\rho_{0}}{\gamma}\Delta T=-\frac{\gamma_{e}\varepsilon_{0}}{2}\Delta E^{2}$,

$\rho_{0}C_{v}\frac{\partial T}{\partial t}-\lambda\Delta T-\frac{C_{v}\left(\gamma-1\right)}{\beta}\frac{\partial\rho}{\partial t}=nc\alpha\varepsilon_{0}E^{2}$.

The electromagnetic variables are under the international system of units (I.U.). Δ is the Laplace operator. *ε*_0_ is the vacuum dielectric constant. The variables *ρ* and *T* in the formula are functions of time and space. ρ is the deviation of density from its average value *ρ*_0_, and *T* is the temperature deviation. *v*, *β*, *η*, and *λ* are the low-frequency limit of the sound velocity, thermal expansion coefficient, viscosity, and heat conductivity, respectively. *γ* is the ratio of the specific heat C_p_ at constant pressure to the specific heat C_v_ at constant volume. *γ_e_* is the electrostriction constant expressing the coupling magnitude between light and elastic strain through the photoelastic effect. *n* is the refractive index, *α* is the absorption coefficient of the light by the material and *c* is the light velocity in vacuum. In the above expressions, viscous fluid is assumed and the changes in the local temperature and density are assumed to be small. *E* is the sum of the electric fields of the light. Eq. is the result of the derivation of the continuity equation and the Navier-Stokes equation [1,2], and the term on the right represent the electrostrictive force. Eq. is the energy-transport equation and the term on the right represent the heat source.

Probe lights are two continuous beams with the same light source, which do not excite Brillouin oscillation in the sample, so the probe lights are not considered in the acoustic wave generation. In the plane-wave approximation, *E* is expressed as the sum of two pump beams:

$E\left(r,t\right)=\sum_{i=1}^{2} A_{i}\left(t-\tau_{i}\right)\exp\left\{i\left[k_{i}\cdot r-\omega_{1}\left(t-\tau_{i}\right)\right]\right\}+c.c.$,

where two pump pulses delayed by *τ_1_* and *τ_2_* are assumed to have the same angular frequency, but have different wave vectors ***k_1_*** and ***k_2_***. The variation of electric field amplitude can be ignored when the light interacts with the sample These two pulses cross in the material with an angle of *2**θ*. The polarization direction of the two pump beams is the same. The light intensity is defined as:

$I_{i}=2n\varepsilon_{0}cA_{i}A_{i}^{*}$.

From Eq. and Eq., it can be found that the driving term is proportional to the intensity of the interference fringe. From Eq. , we get:

$$\begin{matrix} E^{2}=2A_{1}\left(t-\tau_{1}\right)\cdot A_{1}^{*}\left(t-\tau_{1}\right)+2A_{2}\left(t-\tau_{2}\right)\cdot A_{1}^{*}\left(t-\tau_{2}\right) \\ +\left[2A_{1}\left(t-\tau_{1}\right)\cdot A_{2}^{*}\left(t-\tau_{2}\right)\exp\left(ik_{B}\cdot r\right)+c.c.\right] \end{matrix}$$

.

The first two terms of Eq. are spatially independent, they only cause the same temperature increase within the region of interest of the sample and do not produce phonons, so these two terms are not considered later. The interference pattern wave vector generated by superposition is ***k_B_=k_1_-k_2_***,|***k_B_***|*=2ksinθ.*

In the plane-wave approximation, we write:

$\rho\left(r,t\right)=\left[\rho_{1}\left(t\right)\exp(ik_{B}\cdot r)+c.c.\right]$,

.

The overall temperature shift is not considered. Note that the spatial wave vector of *ρ* and *T* is determined to contain only ***k_B_*** and -***k_B_***, because the pump excitation volume is generally larger than the probe detection volume, and the intensity of interference fringes is uniform near the detection volume. The same approach is also adopted in Ref. [2] to solve transient solutions.

Introducing Eq. , , and into Eq. and Eq. , we get:

$\frac{\partial^{2}\rho_{1}}{\partial t^{2}}+\frac{\eta}{\rho_{0}}k_{B}^{2}\frac{\partial\rho_{1}}{\partial t}+v^{2}k_{B}^{2}\rho_{1}=\gamma_{e}\varepsilon_{0}k_{B}^{2}A_{1}\left(t-\tau_{1}\right)A_{2}^{*}\left(t-\tau_{2}\right)-v^{2}\beta\rho_{0}k_{B}^{2}T_{1}$, $\rho_{0}C_{v}\frac{\partial T_{1}}{\partial t}+\lambda k_{B}^{2}T_{1}=2nc\alpha\varepsilon_{0}A_{1}\left(t-\tau_{1}\right)A_{2}^{*}\left(t-\tau_{2}\right)$.

We don’t take the approach of Mountain et al to solve the equation [1]. To simplify the calculation, assume that *γ*-1 is 0. For liquids like water, *γ* is about 1.009. Eq. can be reduced to a Debye relaxation equation. Under this assumption, the temperature is solved as:

$T_{1}\left(t\right)=\frac{2nc\alpha\varepsilon_{0}}{\rho_{0}C_{v}}G_{a}\left(t\right)\otimes\left[A_{1}\left(t-\tau_{1}\right)A_{2}^{*}\left(t-\tau_{2}\right)\right]$,

.

*G_T_(t)* is the green’s function of Debye relaxation equation [3]. H(t) is the unit step function. *Γ_R_=λk_B_*^2^/(*ρ_0_C_v_*) is the temperature relaxation rate, indicating that the spatially periodic temperature distribution, i.e., thermal grating, remains after the propagation of the phonon and then becomes obscure by the heat diffusion. Since the phonon generation through the photothermal mechanism is always accompanied by the thermal grating and also the decay rate of the thermal grating is generally small compared with that of the phonon. Here we do not consider the transfer of absorbed energy to heat. Eq. is a forced harmonic oscillator equation, which green’s function is [3]:

$G_{\rho}\left(t\right)=H(t)\frac{\exp\left(-\Gamma_{B}t\right)\sin\left(\omega_{b}t\right)}{\omega_{b}}$.

*Γ_B_=ηk_B_*^2^/(*2ρ_0_*) is the damping constant of the phonon. *ω_b_* =*(k_B_^2^v^2^-Γ_B_^2^)^1/2^* is the angular frequency. *ρ*_1_ can be expressed as:

$\begin{matrix} \rho_{1}=\gamma_{e}\varepsilon_{0}k_{B}^{2}G_{\rho}\left(t\right)\otimes A_{1}\left(t-\tau_{1}\right)A_{2}^{*}\left(t-\tau_{2}\right) \\ -\frac{2nc\alpha\varepsilon_{0}v^{2}\beta}{C_{v}}k_{B}^{2}G_{\rho}\left(t\right)\otimes\left[G_{a}\left(t\right)\otimes A_{1}\left(t-\tau_{1}\right)A_{2}^{*}\left(t-\tau_{2}\right)\right] \end{matrix}$.

The rightmost item of the above formula can be rearranged. We calculate the convolution of two Green's functions and get:

$\begin{matrix} G_{\rho}\otimes G_{T}=H\left(t\right)\left[\frac{\exp\left(-\Gamma_{R}t\right)-\exp\left(-\Gamma_{B}t\right)\cos\left(\omega_{b}t\right)-\frac{\Gamma_{B}-\Gamma_{R}}{\omega_{b}}\exp\left(-\Gamma_{B}t\right)\sin\left(\omega_{b}t\right)}{\omega_{b}^{2}}\right] \\ \approx H\left(t\right)\left[\frac{\exp\left(-\Gamma_{R}t\right)-\exp\left(-\Gamma_{B}t\right)\cos\left(\omega_{b}t\right)}{\omega_{b}^{2}}\right] \end{matrix}$.

Introducing Eq. and Eq. into Eq. , the *ρ*_1_ can be expressed as:

$\rho_{1}=\frac{\varepsilon_{0}k_{B}^{2}}{\omega_{b}}L\left(t\right)\otimes\left[A_{1}\left(t-\tau_{1}\right)A_{2}^{*}\left(t-\tau_{2}\right)\right]$.

*L*(*t*) is a response function [4] and is expressed as:

$$L=-\gamma_{a}\left[\exp\left(-\Gamma_{R}t\right)-\exp\left(-\Gamma_{B}t\right)\cos\left(\omega_{b}t\right)\right]+\gamma_{e}\exp\left(-\Gamma_{B}t\right)\sin\left(\omega_{b}t\right)$$

*γ_a_* expresses the contribution of the photothermal effect to the dielectric change and is expressed as *γ_a_ =2ncαv^2^β/(C_p_ω_b_)*. The first and second terms of Eq. (17) correspond to the photothermal and photoelastic contributions. The photothermal term is characterized by the coexistence of the relaxational term and the damped harmonic oscillation. For room temperature water, *β=*0.00021/T, *λ=*0.6W/(m∙K), *v*=1500m/s, *C_p_*=4180J/(Kg∙K), *γ_e_*=0.82. The pump uses visible light, *α*<10^-2^/cm. It can be calculated that *γ_a_<<γ_e_*. The photoelastic effect is dominant in this case.

We obtain the induced dielectric constant *δε* as:

$\delta\varepsilon\left(r,t\right)=\frac{\gamma_{e}\rho}{\rho_{0}}=\frac{\gamma_{e}\varepsilon_{0}k_{B}^{2}}{\omega_{b}\rho_{0}}\exp\left(ik_{B}\cdot r\right)\cdot L\left(t\right)\otimes\left[A_{1}\left(t-\tau_{1}\right)A_{2}^{*}\left(t-\tau_{2}\right)\right]+c.c.$.

Considering that the pump pulse width (<10ps) is much smaller than the Brillouin oscillation period (hundreds of ps), the product of two pump pulses is simplified as a delta function in the time domain when *τ_1_=τ_2_=t_0_*.

$A_{1}\left(t-t_{0}\right)\cdot A_{2}^{*}\left(t-t_{0}\right)=\frac{\sqrt{I_{pulse1}I_{pulse2}}}{2n\varepsilon_{0}cS_{e}}\delta\left(t-t_{0}\right)$.

*I_pulse1_* and *I_pulse2_* represent the energy of the two pump pulses and are generally equal. *S_e_* is the pump spot area. Introducing Eq. into Eq., we get:

$\begin{matrix} \delta\varepsilon\left(r,t\right)=\frac{\gamma_{e}k_{B}I_{pump}}{4ncv\rho_{0}S_{e}}\exp\left(ik_{B}\cdot r\right)L\left(t\right)+c.c. \\ =\varepsilon_{1}(t)\exp\left(ik_{B}\cdot r\right)+c.c. \end{matrix}$,

.

*I_pump_* is the pump pulses energy sum. *Λ=2π/k_B_* is the phonon wavelength. The results are consistent with Ref. [4].

**References**

[1] R. D. MOUNTAIN, Spectral distribution of scattered light in a simple fluid, Rev. Mod. Phys. 38 (1966) 205–214.

[2] D. Pohl, W. Kaiser, Time-resolved investigations of stimulated Brillouin scattering in transparent and absorbing media: Determination of phonon lifetimes, Phys. Rev. B 1 (1970) 31–43.

[3] Y. Yan, K. A. Nelson, Impulsive stimulated light scattering. i. general theory, The Journal of Chemical Physics 87 (1987) 6240–6256.

[4] S. Kinoshita, Y. Shimada, W. Tsurumaki, M. Yamaguchi, T. Yagi, New high-resolution phonon spectroscopy using impulsive stimulated Brillouin scattering, Review of Scientific Instruments 64 (1993) 3384–3393.
